# Supplementary material for: Low Spike Antibody Levels and Impaired BA.4/5 Neutralization in Patients with Multiple Myeloma or Waldenstrom’s Macroglobulinemia after BNT162b2 Booster Vaccination
Source: Cancers (Basel). 2022 Nov 25;14(23):5816. doi: 10.3390/cancers14235816 (PMC9737406; doi:10.3390/cancers14235816)
Supplement: Supplementary file 1 [file cancers-14-05816-s001.zip › cancers-1980668-supplementary.pdf]

cancers

MDPI

Supplementary Material: Low Spike Antibody Levels and Impaired BA.4/5 Neutralization in Patients with Multiple Myeloma or Waldenstrom's Macroglobulinemia after BNT162b2 Booster Vaccination

Margherita Rosati, Evangelos Terpos, Jenifer Bear, Robert Burns, Santhi Devasundaram, Ioannis Ntanasis-Stathopoulos, Maria Gavriatopoulou, Efstathios Kastritis, Meletios-Athanasios Dimopoulos, George N. Pavlakis and Barbara K. Felber

A

Cohort	HCW	WM	WM	MM	MM	MM
Therapy	NA	ON (BTKi / rituximab) (G1)	OFF (G2)	ON (anti-CD38/ BCMA) (G3)	ON (PI / IMiD) (G4)	OFF (G5)
n	37	10	8	20	20	19

WA1

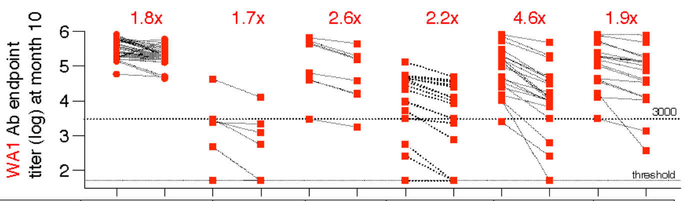

Spike-RBD Ab titer	positive (n)	37	4	8	15	19	19
	>3000 (n)	37	1	7	12	17	17

B

Delta

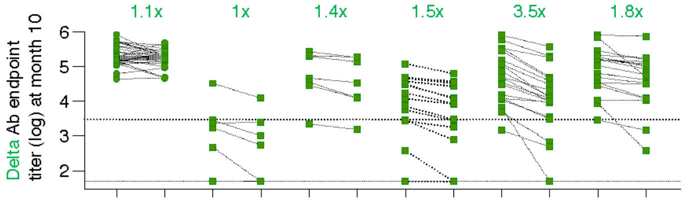

Spike-RBD Ab titer	positive (n)	37	4	8	15	19	19
	>3000 (n)	37	1	7	12	17	17

C

BA.1

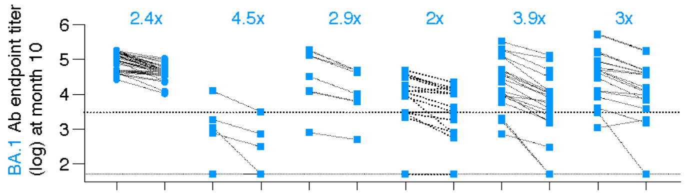

Spike-RBD Ab titer	positive (n)	37	4	8	15	18	19
	>3000 (n)	37	1	7	10	14	16

D

BA.2

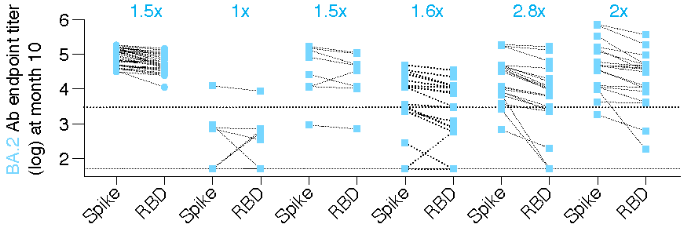

Spike-RBD Ab titer	positive (n)	37	4	8	16	18	19
	>3000 (n)	37	1	7	10	14	17

Figure S1. Comparison of anti-Spike and anti-Spike-RBD Ab. **(A-D)** Comparison of antibody responses to trimeric Spike and Spike-RBD of **(A)** WA1, **(B)** Delta and the Omicron variants **(C)** BA.1 and **(D)** BA.2 in HCW and the different patient cohorts measured after the 3rd dose (month10). The number of samples analyzed; fold difference of antibody titers to Spike and Spike-RBD; the number samples with positive Spike-RBD titers (> threshold 50) and with titers >3000, the threshold applied for sample selection for NAb assay (Fig. 4H), are given.

Figure S2

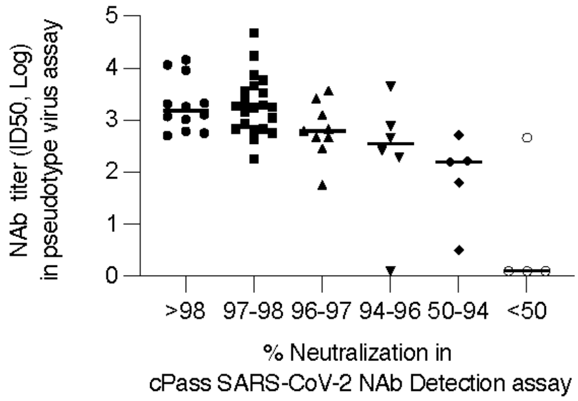

Figure S2. Comparison of NAb using the WA1 pseudotyped neutralization assay and the cPass SARS-CoV2 NAb assay. The data from the cPass assay (x axis) were grouped according to the % neutralization. This comparison shows an overall agreement among the assays and further shows that the pseudotype NAb assay has the power to better distinguish the neutralization capability.
